# Treatment of Recalcitrant Hailey-Hailey Disease With Naltrexone and Dupilumab: A Report of Two Cases

**DOI:** 10.7759/cureus.62701

**Published:** 2024-06-19

**Authors:** Breanna Santoso, Rachel Krevh, Alexa Israeli, Austin Cusick, Dawn Merritt, Michael Holsinger

**Affiliations:** 1 Dermatology, Ohio University Heritage College of Osteopathic Medicine, Dublin, USA; 2 Dermatology, Northeast Ohio Medical University, Rootstown, USA; 3 Medical School, Lake Erie College of Osteopathic Medicine, Bradenton, USA; 4 Dermatology, OhioHealth, Grove City, USA; 5 Dermatology, OhioHealth, Columbus, USA

**Keywords:** treatment-resistant hailey-hailey disease, immunity th2, low-dose naltrexone, dupilumab, hailey-hailey disease

## Abstract

This report presents two cases of patients with long-standing, treatment-resistant Hailey-Hailey disease (HHD) who experienced significant symptom relief through a combination therapy of oral naltrexone and dupilumab injections. The therapeutic potential of targeting the Th2 pathway and Ca^2+^ signaling with dupilumab in managing HHD manifestations is highlighted. The findings suggest that Th2 blockade with dupilumab, in conjunction with naltrexone, effectively controls recalcitrant HHD, indicating a role of cytokine response in altering disease pathogenesis. This case contributes to the growing body of literature on biologic treatments for HHD and suggests avenues for further research in HHD management.

## Introduction

Hailey-Hailey disease (HHD), commonly known as benign familial pemphigus, is a rare autosomal dominant dermatosis characterized by recurrent intertriginous vesicles, erosions, and maceration. Patients with HHD often present with pruritus, burning sensation, pain, body malodor, and psychological distress. Mutation of the ATPase secretory pathway Ca^2+^ transporting 1 (*ATP2C1*) gene leads to changes in calcium (Ca^2+^) concentration within the Golgi apparatus, leading to impaired keratinocyte adhesion and suprabasilar acantholysis [1]. Despite different treatment options, controlling resistant cases of HHD remains difficult.

## Case presentation

Case 1

**Figure 1:**
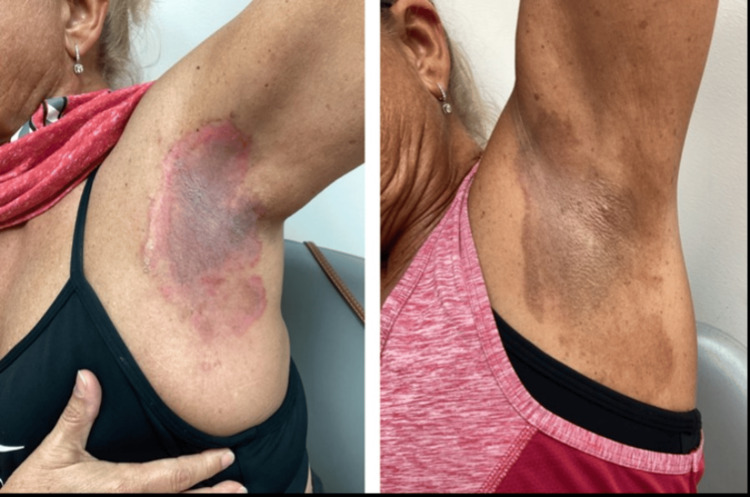
Improvement in Hailey-Hailey disease rash in the left axillary region with naltrexone and dupilumab treatment after 18 months. The first image was taken at the visit of dupilumab initiation.

**Figure 2:**
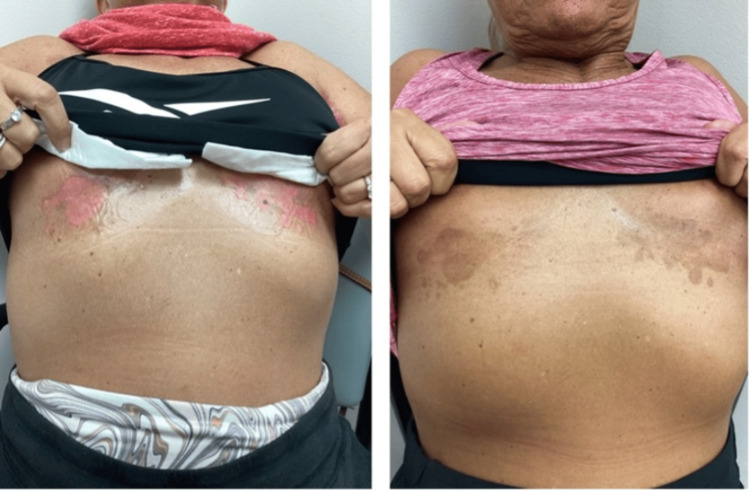
Improvement in Hailey-Hailey disease rash in the inframammary fold with naltrexone and dupilumab treatment after 18 months. The first image was taken at the visit of dupilumab initiation.

A 55-year-old female with a four-year history of painful, pruritic rashes of the axillae, neck, trunk, inner thighs, and vulva was diagnosed with biopsy-proven HHD. Histology revealed characteristic HHD findings, such as acantholysis in a dilapidated brick-wall pattern. The patient had trialed several treatments, including calcipotriene, topical steroids, oral steroids, methotrexate, and Botox injections to control pruritis and pain. Oral naltrexone 1 mg daily was prescribed and later increased to 3 mg daily, which led to only subtle improvement after 16 months of treatment. The patient then received a 600 mg loading dose of dupilumab, followed by a 300 mg maintenance dose every two weeks. Following the addition of dupilumab therapy to the treatment regimen, the patient’s symptomology rapidly improved in a few months. The patient remains clear on a treatment regimen of 300 mg dupilumab every two weeks and 3 mg naltrexone daily. Figure 1 and Figure 2 demonstrate the results of a combined naltrexone and dupilumab regimen over a period of 18 months.

Case 2

A 35-year-old female with a one-year history of a painful, burning rash on the trunk and axillae was diagnosed with biopsy-proven HHD. Multiple treatments, including tacrolimus 0.03% cream, nystatin/triamcinolone cream, and oral naltrexone 1.5 mg were prescribed with minimal sustained treatment response. Naltrexone 1.5 mg daily initially helped but lost efficacy after two months. Dupilumab was added to the treatment regimen, including a 600 mg loading dose followed by a 300 mg maintenance dosing every two weeks. This led to rapid improvement in just four weeks, with a 50% reduction in the body surface area of the rash. The patient became 100% clear five months after dupilumab initiation and remains clear to this day on a combination of 300 mg dupilumab every two weeks with daily 1.5 mg naltrexone.

## Discussion

HHD is caused by loss-of-function mutations in the *ATP2C1* gene, which encodes a calcium pump of the Golgi apparatus involved in a Ca^2+^-dependent signaling pathway [1]. *ATP2C1* is an important regulator of epidermal homeostasis by regulating Notch1 activation of keratinocytes [2]. Notch1 is involved in keratinocyte differentiation and becomes activated in the presence of oxidative stress. When the Ca^2+^ concentration gradient becomes disrupted in HHD, increased oxidative stress downregulates the DNA damage response, impacting keratinocyte differentiation and initiating a cascade of inflammatory mediators [2]. This leads to a loss of cytoskeletal adhesion between adjacent cells by disrupting the structure of cadherins and adhesion junctions, resulting in acantholysis [3].

While there is no current cure for HHD, therapeutic treatment currently aims to manage symptoms by reducing inflammation, controlling exacerbation, and prolonging remissions. First-line treatment for exacerbation of HHD includes moderate to high-intensity topical steroids followed by topical calcineurin inhibitors. The use of corticosteroids regulates pro-inflammatory cytokines interleukin (IL)-6 and IL-8, which indirectly play a role in *ATP2C1* expression and the suppression of T-cell-mediated immune responses [1]. Topical calcineurin inhibitors (tacrolimus 0.1% ointment and pimecrolimus 1% cream applied twice daily) suppress the immune system by binding to the FK506 protein, thus blocking transcription on IL-2 and inhibiting T-cell proliferation [4].

Despite the large variety in treatment, some patients continue to experience recurrent flare-ups and intense symptoms. Naltrexone is a μ-receptor antagonist that treats HHD by antagonizing toll-like receptor 4 (TLR4), which is constitutively expressed in keratinocytes [5]. When TLR4 activity is blocked, calcium homeostasis is maintained and cytokine release is inhibited [5]. This mechanism may play a role in maintaining keratinocyte adhesion and preventing acantholysis.

Dupilumab is commonly used as a treatment for atopic dermatitis, but its anti-inflammatory nature has recently shown remarkable improvement in the treatment of HHD. This human monoclonal antibody works by binding to IL-4 and IL-13 receptors, which share a type II heterodimer receptor complex that triggers Th2 differentiation. Additionally, IL-4 binds to a type I receptor that phosphorylates Janus kinase to activate signal transducer and activator of transcription 6 transcription factor, upregulating inflammatory-mediated genes [6-8]. The role of dupilumab in HHD is unclear. Type 2 inflammation plays a strong role in the humoral immune response and is characterized by the release of IL‐4, IL‐13, and IL‐5 cytokines [7]. Dupilumab’s clinical efficacy suggests that Th2 inflammation may, in part, affect the HHD state.

Additionally, there is evidence that supports dupilumab’s possible role in Ca^2+^ intracellular signaling in keratinocytes. SPARC-related modular calcium-binding protein 1 is involved in the regulation of keratinocyte differentiation and is an early target suppressed by IL-4 and IL-13 through its extracellular Ca^2+^ binding domain [3]. It helps maintain the Ca^2+^ gradient and keeps the epidermal barrier mature and healthy. The conjugation of dupilumab with naltrexone enhances immunosuppression and maintains Ca^2+^ homeostasis in keratinocytes, leading to positive dermatological outcomes in the treatment of HHD.

## Conclusions

We provide a convincing report of two patients with long-standing, treatment-resistant HHD who achieved significant symptom relief while undergoing a combination of naltrexone and dupilumab injection therapy. While the involvement of the Th2 pathway and Ca^2+^ signaling in HHD are not yet fully understood, the potential therapeutic implications of targeting these pathways with dupilumab warrant further investigation to explore its efficacy in managing the manifestations of HHD. Our case suggests that Th2 blockade using dupilumab, and possibly the addition of naltrexone, provides significant control of recalcitrant HHD. While naltrexone has been linked to HHD clearance in the literature, our evidence of naltrexone-associated disease clearance portrayed in these studies is not directly correlated, as clearance was sustained after the addition of dupilumab. This case contributes to the emerging literature on biologic use in HHD and proposes research targets for further HHD management.
